# Determinants of health insurance enrolment in Ghana: evidence from three national household surveys

**DOI:** 10.1093/heapol/czz079

**Published:** 2019-08-21

**Authors:** Paola Salari, Patricia Akweongo, Moses Aikins, Fabrizio Tediosi

**Affiliations:** 1 Department of Epidemiology and Public Health, Swiss Tropical and Public Health Institute (Swiss TPH), Socinstrasse 57, Basel, Switzerland; 2 University of Basel, Basel, Switzerland; 3 School of Public Health, College of Health Sciences, University of Ghana, Legon, Accra, Ghana

**Keywords:** Ghana, health insurance, household surveys, NHIS, determinants of enrolment

## Abstract

In 2003, Ghana implemented a National Health Insurance Scheme (NHIS) to move towards Universal Health Coverage. NHIS enrolment is mandatory for all Ghanaians, but the most recent estimates show that coverage stands under 40%. The evidence on the relationship between socio-economic characteristics and NHIS enrolment is mixed, and comes mainly from studies conducted in a few areas. Therefore, in this study we investigate the socio-economic determinants of NHIS enrolment using three recent national household surveys. We used data from the Ghanaian Demographic and Health Survey conducted in 2014, the Multiple Indicator Cluster Survey conducted in 2011 and the sixth wave of the Ghana Living Standard Survey conducted in 2012–13. Given the multilevel nature of the three databases, we use multilevel logistic regression models to estimate the probability of enrolment for women and men separately. We used three levels of analysis: geographical clusters, household and individual units. We found that education, wealth, marital status—and to some extent—age were positively associated with enrolment. Furthermore, we found that enrolment was correlated with the type of occupation. The analyses of three national household surveys highlight the challenges of understanding the complex dynamics of factors contributing to low NHIS enrolment rates. The results indicate that current policies aimed at identifying and subsidizing underprivileged population groups might insufficiently encourage health insurance enrolment.

**Key Messages**

- Education, wealth, marital status and occupation were associated with enrolment.
- Women and men working in the agricultural sector, manual workers, casual workers, domestic employees and those in the retail sale sector were less likely to be enrolled in the NHIS than individuals who were not employed.
- Policy makers should re-design the policies to identify and subsidize the worst off.

## Introduction 

In low- and middle-income countries (LMIC) one of the obstacles for Universal Health Coverage (UHC) is including vulnerable populations in social health protection schemes such as social health insurance (WHO, 2010, 2013). In 2003, the government of Ghana implemented a single National Health Insurance Scheme (NHIS) that requires all Ghanaians to enrol either into the NHIS, or into one of the private health insurance schemes. Each of these schemes are required to provide basic health care benefits as delineated by the National Health Insurance Authority. However, after more than 10 years, <40% of the population is enrolled in the NHIS (Agyepong *et al.*, 2016).

NHIS is financed through a number of sources: 70% through indirect tax (2.5% levy on selected goods and services), 20–25% through social security contributions of formal workers (Social Security and National Insurance Trust—SSNIT—contributors), <5% through the annual contributions from the subscribers and a small percentage from development partners (Nguyen *et al.*, 2011). The NHIS yearly premium ranges from GHS 7.20 (USD 1.60) to GHS 48.00 (USD 10.60) per person, depending on the region of residence (http://www.nhis.gov.gh/faqs.aspx). In addition, all members must pay an initial membership card processing fee of GHS 8.00 (USD 1.82) and GHS 5.00 (USD 1.14) for yearly renewal. The benefits package secured by NHIS should cover over 95% of disease conditions (http://www.nhis.gov.gh/benefits.aspx). The health services included are listed in the NHIS website: http://www.nhis.gov.gh/benefits.aspx.

Some populations receive premium exemptions: children below the age of 18 years, the elderly aged 70 years or above, SSNIT pensioners, pregnant women, indigents, people with mental disorders, differently abled people and beneficiaries of the Livelihood Empowerment Against Poverty (LEAP) programme. Some of these categories are also exempted from the yearly processing fee: pregnant women, indigents and LEAP beneficiaries (http://www.nhis.gov.gh/about.aspx). Despite the mandatory nature of the NHIS and numerous premium exemptions, many people have either never registered or fail to renew their NHIS membership. Unregistered individuals have to make out-of-pocket payments to access health services, potentially incurring financial hardship (Akazili *et al.*, 2017).

Several studies have assessed the relationship between individual or households’ socio-economic characteristics and the decision to enrol in social health insurance plans in LMIC. Most often, low-income and low-educational attainment were correlated with low enrolment rates (Sinha *et al.*, 2007; Adebayo *et al.*, 2015; Panda *et al.*, 2016; Dror *et al.*, 2016) or high dropout rates (Atinga *et al.*, 2015). In some studies being male, young and in a larger household were also correlated with enrolment in social health insurance (Adebayo *et al.*, 2015; Sarker *et al.*, 2017), but these findings were not confirmed by other studies (Panda *et al.*, 2014). Additionally, a lack of trust in the insurance scheme and a perception of poor health care quality were associated with lower enrolment rates (Criel and Waelkens, 2003; Jehu-Appiah *et al.*, 2012; Adebayo *et al.*, 2015).

Similarly, studies focusing on Ghana have not reached a consensus on the main determinants of NHIS enrolment, and most of the evidence comes from studies conducted in selected areas (Sarpong *et al.*, 2010; Gobah and Zhang, 2011; Jehu-Appiah *et al.*, 2011; Boateng and Awunyor-Vitor, 2013; Atinga *et al.*, 2015; Duku *et al.*, 2015).

The aim of this study is to identify which socio-economic and demographic characteristics are associated with NHIS enrolment. While previous studies used data from a few geographic areas, we use three different household surveys designed to be nationally representative. Our approach is also novel in terms of its empirical strategy. We estimate multilevel models including small geographic areas as random effects, thus assessing not only demand-side factors, but also accounting for the heterogeneity attributable to regional and local health systems and other contextual factors.

## Data

We used three nationally representative household surveys: the Demographic and Health Survey (DHS), the Multiple Indicators Cluster Surveys (MICS) and the Ghana Living Standard Survey (GLSS). These surveys collect information on the main socio-economic household characteristics and on individual NHIS enrolment status. They all have been designed to be representative at both national and regional levels.

### Demographic and Health Survey

The ongoing DHS programme has collected data from over 90 countries and in Ghana, the first wave was collected in 1988 and new waves were collected at regular intervals every 5 years. The DHS survey focuses on population and household characteristics, health, nutrition and lifestyle with special emphasis on topics that affect the lives of children and women, including fertility and childhood mortality levels, fertility preferences and family planning methods. We used the latest wave of data available to us, collected in Ghana in 2014, which included 12 831 households (Ghana Statistical Service, Ghana Health Service, and ICF International, 2015).

### Multiple Indicators Cluster Surveys

The MICS have been ongoing since the mid-1990s and are available for >100 countries. MICS are specifically focused on children and women, and include, like the DHS, information on the use of various types of health services and health outcomes, as well as a comprehensive set of socio-economic characteristics. We used the most recent survey, implemented in 2011, which includes 12 150 households, the majority of which were individual interviews with women (10 963 women and 3511 men individually interviewed) (Ghana Statistical Service, 2012).

### Ghana Living Standard Survey

The GLSS is a household survey designed to provide a broad picture of the living conditions in the country, including households’ consumption and expenditure. We used the most recent survey implemented in 2012–13 that collected data on 18 000 households (Ghana Statistical Service, 2012).

### Sampling design

The three surveys used a similar multi-stage sampling approach. Specific enumeration areas were selected to form the Primary Sample Unit (427 areas in the DHS, 810 in the MICS and 1200 in the GLSS). The enumeration areas were stratified by the 10 administrative regions at the first level and then by urban–rural area within each region at the second level. For each enumeration area, a specific number of households represented the Secondary Sample Unit (Ghana Statistical Service, 2012, 2014; Ghana Statistical Service, Ghana Health Service, and ICF International, 2015).

## Analysis

We used multilevel logistic regression models to assess the association between NHIS enrolment and socio-economic characteristics of the population, controlling for the geographical areas. We analysed men and women separately because we are interested in assessing possible difference in their behaviour. The dependent variable of the analysis was equal to one if the individual reported to be registered with the NHIS and zero otherwise. In the main analysis, only the respondents who could show the insurance card to the enumerator were considered enrolees. In Supplementary Material, we report the results including in the dependent variable the people who reported to be registered in the NHIS but could not show the card.

We examined different specifications to assess the robustness of the model. In particular, we first ran the model keeping the whole sample, and then we excluded pregnant women and people under 18 years of age, the largest groups that receive premium exemptions. We were unable to identify beneficiaries of the LEAP contribution, the people enrolled in the NHIS as indigents, SSNIT contributors, SSNIT pensioners or people affected by mental illnesses eligible for premium exemptions. We included as explanatory variables all individual and household characteristics expected to influence the NHIS enrolment, regional dummy variables and urban/rural dummy variables.

We ran a multilevel regression model, where the enumeration area represented the first level, the household the second level and the individual the third level of analysis. In this way, the estimates accounted for the variability across geographical areas related to unobserved contextual factors, including those related to the health services such as the presence and accessibility of health care providers, the quality of the services, and other fixed effects/characteristics of each particular area. Furthermore, this approach allowed us to account for any residual correlation within households. While there is consensus in the literature that sample weights should be used in descriptive statistics (Kish and Frankel, 1974; Solon *et al.*, 2015), the use of weights in hierarchical models is much more controversial (Gelman, 2007; Bollen *et al.*, 2016). For this reason, sample weights were applied only in the percentage values of the descriptive statistics (Table 1).

**Table 1:** Descriptive statistics for people enrolled in NHIS

	DHS (2014)		MICS (2011)		GLSS (2012–13)	
	Women (%)	Men (%)	Women (%)	Men (%)	Women (%)	Men (%)
Marital status						
Not married	29.1	29.9	21.6	17.8	33.3	27.9
Married	38.4	32.0	31.3	20.2	35.7	32.1
Sex of household head						
Female	31.3	30.5	24.8	20.0	33.3	31.0
Male	36.3	31.1	29.0	18.9	36.1	31.3
Children at home						
No	30.4	29.3	23.6	21.9	34.4	32.1
Yes	36.8	33.4	29.9	20.1	36.6	28.9
Pregnant						
No	33.1		26.8		39.3	
Yes	50.2		36.6		48.2	
Age classes						
15–19 (<20 for GLSS)	31.3	34.7	23.1	22.7	34.6	34.8
20–24	29.7	26.7	27.2	11.2	31.1	27.8
25–29	37.0	22.4	27.2	19.4	34.4	21.3
30–34	38.4	26.1	32.2	18.0	33.3	24.2
35–39	39.0	36.0	29.6	21.7	37.1	24.1
40–44	32.7	33.0	26.8	18.0	36.3	28.2
45–49	32.5	37.3	28.2	16.2	35.1	28.4
50–54		32.0		24.6	37.0	29.1
55–59		37.5		19.6	42.5	37.4
Wealth index						
Poorest	32.9	35.6	21.5	14.4	32.8	25.7
Poorer	36.7	26.3	25.5	20.9	33.1	28.6
Middle	35.2	25.7	25.6	15.3	35.7	31.0
Richer	33.4	30.6	30.3	19.6	35.1	32.8
Richest	33.9	35.9	32.3	23.2	37.8	35.6
Education						
No education	33.5	34.2	24.6	16.9	33.9	30.3
Primary	33.5	23.6	24.0	15.6		
Middle	34.8	29.3	27.5	17.9		
Secondary/higher	35.0	37.1	34.8	23.2		
Primary not completed					32.8	31.0
Completed primary					30.0	30.0
Lower secondary					35.0	26.7
Upper secondary					45.7	29.8
Post-secondary					42.4	33.1
University and higher					38.8	40.6
Having hypertension						
No	34.1	29.9				
Yes	37.5	49.7				
Morbidity						
No						31.0
Yes						32.8
Disability						
No					35.1	31.2
Yes					36.1	35.8
Occupation						
Not working	34.9	39.7			35.2	34.6
Professional/technical/managerial	38.5	40.8				
Clerical	45.6	37.0				
Sales	33.6	26.6				
Agriculture	34.5	31.6				
Services	36.7	42.6				
Skilled manual	32.2	23.9				
Unskilled manual	34.8	21.9				
Paid employee					36.4	28.6
Non-agric s.e. with employees					36.3	28.2
Non-agric s.e. without employees					35.1	24.9
Non-agric family worker					32.6	35.9
Agric s.e. with employees					32.3	29.9
Agric s.e. without employees					36.9	26.3
Agric family worker					34.5	32.6
Domestic employee					25.2	13.1
Casual workers					44.4	18.6
Type of place of residence						
Rural	35.3	28.6	28.0	19.0	34.8	30.9
Urban	33.5	33.3	27.3	19.2	35.5	31.7
Regions						
Western	39.8	27.3	24.1	19.1	24.4	23.7
Central	23.4	19.2	13.1	9.4	27.8	24.5
Greater Accra	26.1	18.1	15.9	8.5	28.9	24.7
Volta	54.0	31.7	25.5	11.6	38.1	32.2
Eastern	34.9	26.2	28.1	23.1	39.0	31.8
Ashanti	33.3	37.6	42.6	27.0	30.9	27.8
Brong Ahafo	48.9	46.9	34.7	29.2	49.2	43.9
Northern	29.4	42.6	21.1	14.2	33.1	30.8
Upper east	31.1	55.7	33.7	19.7	59.7	56.8
Upper west	40.3	40.9	42.5	30.3	58.8	54.7

DHS 2014, MICS, 2011 and GLSS 2012–13.

s.e., ‘self-employed’.

Supplementary Table S6 contains a detailed description of all the variables included in the regression models. The analyses were run using STATA 14.0.

## Results

### Descriptive statistics

#### Data on enrolment

The percentage of respondents enrolled in the NHIS in the three surveys are shown in Table 2. The three surveys included three different possible answers to the question regarding enrolment in the NHIS: ‘no’, ‘yes card seen’ in case the person was able to show a valid card during the interview and ‘yes, card not seen’ when the person was not able to show the card to the enumerator. According to DHS data, 35.5% of the women and men in 2014 had a valid insurance card and were able to show it to the enumerator. Although some people who reported membership and were unable to show their insurance card may have actually hold a valid insurance card, the official statistics and key informants indicated that the majority of them may either have had an expired card or erroneously believe to be enrolled. Women (22.5%) were more likely than men (9.2%) to be unable to show their insurance card despite responding that they were enrolled. In the MICS, the proportion of members of households enrolled was smaller than that reported by the DHS; only 27.6% of women and 18.8% of men were able to show a valid insurance card, while 13.2% of women and 13.0% of men reporting having it but were unable to show it. According to the GLSS data 38.1% of women and 34.4% of men were able to show a valid NHIS card in 2012–13.

**Table 2:** Number of people holding a valid insurance card DHS, MICS and GLSS

	Women		Men		Total	
Hold a valid NHIS card	*N*	%	*N*	%	*N*	%
DHS (2014)						
No	3915	41.8	2432	55.7	6347	46.2
Yes, card seen	3347	35.7	1528	35.0	4875	35.5
Yes, card not seen	2110	22.5	403	9.2	2513	18.3
Total	9372	100	4363	100	13 735	100
MICS (2011)						
No	6225	59.1	2249	68.1	8474	61.3
Yes, card seen	2914	27.6	621	18.8	3535	25.6
Yes, card not seen	1391	13.2	430	13.0	1821	13.2
Total	10 530	100	3300	100	13 830	100
GLSS (2012–13)						
No	15 892	43.3	17 002	49.1	32 894	46.1
Yes, card seen	13 998	38.1	11 896	34.4	25 894	36.3
Yes, card not seen	6828	18.6	5683	16.4	12 511	17.5
Total	36 718	100	34 581	100	71 299	100

#### Sample characteristics

Table 1 shows descriptive statistics for DHS, MICS and GLSS data, for people enrolled and able to show a card. In all the three databases the percentage of people enrolled was slightly higher for women living in households with a male head-of-household compared with women in households with a female head-of-household (36.3% vs 31.1% in the DHS, 29% vs 24.8% in the MICS and 36.1% vs 33.3% in the GLSS), and the difference was smaller among men. Married women reported a higher percentage of NHIS enrolment (38.4%, 31.3% and 35.7%) than unmarried women (29.1%, 21.6% and 33.3%), while among men the enrolment did not change significantly with marriage. Similarly, women with at least one child (and also men in the DHS sample) reported a higher percentage of NHIS enrolment. In the DHS and GLSS samples around half of pregnant women were registered compared with 33% of non-pregnant women (39.3 for GLSS).

Age classes show slightly different patterns of enrolment across the three databases. While overall higher levels of wealth and education showed higher enrolment rates, there were no remarkable differences in the sample of DHS women.

Only 34.5% of women and 31.6% of men employed in the agricultural sector were enrolled in the NHIS, compared with around 40% of the clerics and people in the service sector and people in the category ‘professional/technical/managers’. Skilled and unskilled manual workers showed the lowest enrolment rate for both men (32.2% and 34.8%, respectively) and women (23.9% and 21.9%, respectively). In the GLSS sample, women employed as casual workers tended to register more than the average (44.4%), while the category with the smallest percentage of women registered was the ‘domestic workers’ (25.2%), similarly to men (13.1%). Unemployed men, together with those who were ‘family workers’ both in agricultural and non-agricultural settings, reported the highest enrolment rates among men.

Close to 50% of men who were diagnosed with hypertension were enrolled in the NHIS, while this percentage was lower for women. Men with a disability reported a slightly higher enrolment rate (35.8%) than those without (31.2%). As far as geographical differences are concerned, the percentage of people enrolled did not seem to remarkably change between urban or rural areas.

The regions with the highest proportion of people registered were the Upper West, Volta and Brong Ahafo regions (and also Upper East in the MICS and GLSS samples and Ashanti in the MICS sample).

### Results from the regression model

#### Demographic Health Surveys

Regression results obtained with DHS data are found in Table 3, including results obtained with the whole sample, as well as results excluding pregnant women and people under 18 years of age, groups that are exempted from NHIS premiums.

**Table 3:** OR from a multilevel logistic regression

	Whole sample		Exempted excluded	
Prob. of holding a valid NHIS card	Women	Men	Women	Men
Married	1.668∗∗∗	1.315	1.583∗∗∗	1.318
	[1.390–2.003]	[0.989–1.748]	[1.313–1.907]	[0.998–1.739]
Male head	1.041	1.124	1.082	1.167
	[0.886–1.224]	[0.821–1.539]	[0.912–1.283]	[0.815–1.671]
Children <5	1.123∗∗	1.110	1.168∗∗∗	1.097
	[1.040–1.213]	[0.993–1.242]	[1.080–1.265]	[0.978–1.231]
Pregnant	2.610∗∗∗			
	[2.055–3.317]			
Age 15–17	1.332∗	1.837∗∗∗		
	[1.055–1.682]	[1.320–2.555]		
Age 18–24	1	1	1	1
Age 25–29	1.342∗∗	0.551∗∗∗	1.363∗∗	0.541∗∗∗
	[1.090–1.653]	[0.388–0.784]	[1.101–1.686]	[0.383–0.765]
Age 30–34	1.485∗∗∗	0.723	1.584∗∗∗	0.712
	[1.188–1.857]	[0.488–1.073]	[1.259–1.992]	[0.485–1.044]
Age 35–39	1.533∗∗∗	1.428	1.703∗∗∗	1.373
	[1.216–1.933]	[0.957–2.130]	[1.344–2.158]	[0.930–2.026]
Age 40–44	1.332∗	1.160	1.508∗∗	1.133
	[1.038–1.710]	[0.769–1.750]	[1.173–1.940]	[0.760–1.689
Age 45–49	1.352∗	1.909∗∗	1.517∗∗	1.815∗∗
	[1.036–1.765]	[1.234–2.952]	[1.165–1.976]	[1.186–2.779]
Age 50–54		1.940∗∗		1.833∗∗
		[1.233–3.051]		[1.178–2.852]
Age 55–59		1.601		1.545
		[0.983–2.606]		[0.960–2.488]
Poorest	1	1	1	1
Poorer	1.287∗	1.119	1.142	1.211
	[1.008–1.644]	[0.808–1.551]	[0.887–1.470]	[0.869–1.688]
Middle	1.295	1.273	1.226	1.369
	[0.975–1.720]	[0.873–1.856]	[0.916–1.643]	[0.929–2.017]
Richer	1.330	2.023∗∗	1.226	2.395∗∗∗
	[0.963–1.838]	[1.301–3.144]	[0.878–1.710]	[1.514–3.788]
Richest	1.816∗∗	2.782∗∗∗	1.686∗∗	3.214∗∗∗
	[1.253–2.631]	[1.673–4.626]	[1.152–2.468]	[1.896–5.447]
No education	1	1	1	1
Primary education	1.047	0.982	1.081	1.036
	[0.853–1.284]	[0.696–1.386]	[0.873–1.339]	[0.728–1.475]
Middle education	1.187	1.323	1.282∗	1.321
	[0.977–1.443]	[0.968–1.807]	[1.047–1.568]	[0.967–1.806]
Secondary education	1.348∗	2.186∗∗∗	1.438∗∗	2.190∗∗∗
	[1.043–1.742]	[1.491–3.206]	[1.102–1.876]	[1.489–3.221]
Not working	1.529∗∗∗	2.116∗∗∗	1.546∗∗∗	1.872∗∗
	[1.227–1.905]	[1.493–3.000]	[1.216–1.965]	[1.243–2.817]
Professional sector	2.199∗∗∗	1.775∗∗	2.350∗∗∗	1.639∗
	[1.543–3.135]	[1.200–2.626]	[1.630–3.386]	[1.107–2.427]
Clerical sector	2.024∗	1.822	2.197∗	1.654
	[1.077–3.804]	[0.832–3.990]	[1.171–4.122]	[0.771–3.547]
Sales	1.445∗∗∗	1.079	1.368∗∗	0.968
	[1.177–1.774]	[0.718–1.622]	[1.105–1.694]	[0.641–1.461]
Agriculture	1	1	1	1
Services	2.145∗∗	1.606	2.065∗∗	1.505
	[1.277–3.603]	[0.862–2.994]	[1.210–3.525]	[0.807–2.809]
Skilled manual	1.306∗	0.876	1.291∗	0.816
	[1.020–1.671]	[0.634–1.209]	[1.002–1.664]	[0.587–1.134]
Unskilled manual	1.403	0.980	1.255	0.920
	[0.808–2.438]	[0.686–1.400]	[0.705–2.235]	[0.642–1.318]
Hypertension	1.203	1.114	1.169	1.165
	[0.942–1.538]	[0.896–1.385]	[0.917–1.489]	[0.927–1.464]
Urban	0.869	1.147	0.870	1.089
	[0.661–1.143]	[0.833–1.581]	[0.663–1.142]	[0.791–1.500]
Western	0.485∗∗	0.233∗∗∗	0.591∗	0.264∗∗∗
	[0.298–0.788]	[0.136–0.398]	[0.367–0.950]	[0.155–0.450]
Central	0.191∗∗∗	0.119∗∗∗	0.206∗∗∗	0.131∗∗∗
	[0.115–0.317]	[0.0667–0.213]	[0.124–0.342]	[0.0724–0.238]
Greater Accra	0.176∗∗∗	0.0628∗∗∗	0.188∗∗∗	0.0749∗∗∗
	[0.105–0.295]	[0.0339–0.116]	[0.112–0.314]	[0.0401–0.140]
Volta	1.247	0.399∗∗∗	1.258	0.439∗∗
	[0.758–2.052]	[0.231–0.689]	[0.772–2.048]	[0.256–0.752]
Eastern	0.398∗∗∗	0.186∗∗∗	0.426∗∗∗	0.179∗∗∗
	[0.244–0.648]	[0.108–0.322]	[0.263–0.690]	[0.103–0.313]
Ashanti	0.317∗∗∗	0.380∗∗∗	0.341∗∗∗	0.396∗∗∗
	[0.194–0.518]	[0.225–0.641]	[0.210–0.553]	[0.236–0.664]
Brong Ahafo	1	1	1	1
Northern	0.392∗∗∗	0.794	0.425∗∗	0.924
	[0.234–0.657]	[0.463–1.359]	[0.255–0.710]	[0.546–1.563]
Upper east	0.429∗∗	2.252∗∗	0.519∗	2.448∗∗
	[0.254–0.725]	[1.293–3.921]	[0.309–0.872]	[1.410–4.250]
Upper west	0.837	0.797	0.841	0.918
	[0.491–1.425]	[0.455–1.398]	[0.498–1.420]	[0.530–1.589]
*N*	9339	4348	7579	3818

Women and men, DHS 2014.

Odds ratios; 95% confidence intervals in brackets.

* *P < *0.05;

** *P < *0.01;

*** *P < *0.001.

Among women, being married was positively correlated with enrolment (OR = 1.668, 99% CI: 1.390–2.003), as well as having children under 5 years old (OR = 1.123, 99% CI: 1.040–1.213). Marital status may have captured the effect of having a male as head-of-household, a variable which was not statistically significant in the presence of the marital status indicator. As expected, pregnant women were more likely to be registered in the NHIS (OR = 2.610, 99% CI: 2.055–3.317).

There were no large differences in registration rates by age. Using the group aged 18–24 years as the referent, people under 18 years old were more likely to be registered (OR = 1.332, 95% CI: 1.055–1.682), potentially due to the premium exemption afforded to minors (although a registration fee still applies). Among women, all other age groups were more likely to be registered than people aged 18–24 years, especially those aged 35–39 years old (OR = 1.533, 95% CI: 1.216–1.933). Men aged 25–29 years old were least likely to register (OR = 0.551, 99% CI: 0.388–0.784), while the elderly (over 45 years) were more likely to be enrolled than the reference category (OR = 1.909, 99% CI: 1.234–2.952).

Women and men with secondary education (OR = 1.348, 95% CI: 1.043–1.742 and OR = 2.186, 99% CI: 1.491–3.206, respectively) or of the highest wealth category (OR = 1.816, 99% CI: 1.253–2.631 and OR = 2.78, 99% CI: 1.673–4.626, respectively) were both associated with a higher probability of being enrolled in the NHIS. For men, the wealth effect was positive and significant for the second richest wealth category as well (OR = 2.023, 99% CI: 1.301–3.144).

Women (OR = 2.199, 99% CI: 1.543–3.135) and men (OR = 1.775, 99% CI: 1.200–2.626) working in the professional sector were more likely to be enrolled in the NHIS than people employed in the agricultural sector. Also skilled-manual women (OR = 1.306, 95% CI: 1.020–1.671) as well as those employed in the sales (OR = 1.445, 99% CI: 1.177–1.774) or services sector (OR = 2.145, 99% CI: 1.277–3.603) were more likely to be enrolled than those working in the agricultural sector. Interestingly, even for unemployed men (OR = 2.116, 99% CI: 1.493–3.000) and women (OR = 1.529, 99% CI: 1.227–1.905) the probability of being enrolled was higher than for people employed in the agricultural sector.

There was insufficient evidence that rural or urban residence impacts NHIS enrolment, but the regional variability in enrolment rates shown in the descriptive statistics is confirmed in the regression analysis. The NHIS was first piloted and implemented in the Brong Ahafo region where the level of enrolment remains one of the highest. We used this region as the referent in the analysis. In most of the other regions, the probability of being enrolled was significantly lower than in Brong Ahafo. The only exceptions were Volta for women, Northern for men and Upper West for both genders, for which results were not statistically significant. Upper East was the only region where men were more likely to enrol than in Brong Ahafo region (OR = 2.25). There are no remarkable differences in the results excluding people with premium exemptions.

#### Multiple Indicator Cluster Surveys

Results obtained with MICS data are shown in Table 4, which are similar to those obtained with DHS data. Among women, enrolment was positively correlated with being married (OR = 1.646, 99% CI: 1.312–2.065) and having children at home (OR = 1.606, 99% CI: 1.288–2.003), consistent with the results of DHS data. In this analysis, being married was positively correlated and statistically significant for men as well (OR = 1.794, 95% CI: 1.140–2.824).

**Table 4:** OR from a multilevel logistic regression

	Whole sample		Exempted excluded	
Prob. of holding a valid NHIS card	Women	Men	Women	Men
Married	1.646∗∗∗	1.794∗	1.548∗∗∗	1.759∗∗
	[1.312–2.065]	[1.140–2.824]	[1.226–1.953]	[1.150–2.691]
Male head	1.143	0.714	1.259∗	0.721
	[0.948–1.378]	[0.440–1.160]	[1.034–1.534]	[0.422–1.234]
Children at home	1.606∗∗∗	1.123	1.482∗∗∗	1.116
	[1.288–2.003]	[0.827–1.525]	[1.178–1.865]	[0.825–1.508]
Pregnant	2.034∗∗∗			
	[1.609–2.572]			
Age 15–17	1.349∗	2.545∗∗∗		
	[1.041–1.748]	[1.609–4.024]		
Age 18–24	1	1	1	1
Age 25–29	1.079	1.093	1.167	1.033
	[0.850–1.370]	[0.659–1.814]	[0.913–1.491]	[0.647–1.651]
Age 30–34	1.309∗	1.136	1.430∗∗	1.097
	[1.020–1.679]	[0.631–2.048]	[1.107–1.848]	[0.638–1.885]
Age 35–39	1.115	1.396	1.185	1.304
	[0.863–1.440]	[0.767–2.542]	[0.912–1.539]	[0.751–2.265]
Age 40–44	1.231	1.055	1.286	1.023
	[0.940–1.612]	[0.549–2.026]	[0.980–1.689]	[0.560–1.867]
Age 45–49	1.071	1.657	1.179	1.568
	[0.813–1.409]	[0.957–2.868]	[0.897–1.549]	[0.943–2.606]
Poorest	1	1	1	1
Poorer	2.378∗∗∗	3.104∗∗∗	2.388∗∗∗	2.871∗∗∗
	[1.854–3.051]	[1.979–4.868]	[1.841–3.098]	[1.855–4.446]
Middle	3.378∗∗∗	2.666∗∗∗	3.337∗∗∗	2.363∗∗
	[2.474–4.612]	[1.507–4.718]	[2.407–4.626]	[1.368–4.084]
Richer	5.382∗∗∗	5.495∗∗∗	5.115∗∗∗	4.629∗∗∗
	[3.806–7.611]	[2.947–10.25]	[3.551–7.366]	[2.557–8.382]
Richest	7.950∗∗∗	9.373∗∗∗	7.646∗∗∗	6.744∗∗∗
	[5.304–11.92]	[4.391–20.01]	[4.997–11.70]	[3.306–13.76]
No education	1	1	1	1
Primary education	1.205	0.834	1.260∗	0.819
	[0.978–1.484]	[0.525–1.323]	[1.009–1.573]	[0.511–1.314]
Middle education	1.443∗∗∗	1.453	1.305∗	1.257
	[1.170–1.781]	[0.965–2.187]	[1.046–1.628]	[0.841–1.878]
Secondary education	2.832∗∗∗	2.225∗∗	2.602∗∗∗	2.216∗∗∗
	[2.134–3.759]	[1.377–3.593]	[1.931–3.507]	[1.395–3.519]
Urban	0.752∗	0.656∗	0.710∗∗	0.689
	[0.586–0.965]	[0.435–0.988]	[0.552–0.914]	[0.466–1.019]
Western	0.296∗∗∗	0.295∗∗	0.253∗∗∗	0.280∗∗∗
	[0.177–0.495]	[0.140–0.623]	[0.151–0.426]	[0.135–0.578]
Central	0.0939∗∗∗	0.102∗∗∗	0.0956∗∗∗	0.134∗∗∗
	[0.0588–0.150]	[0.0490–0.212]	[0.0591–0.155]	[0.0671–0.269]
Greater Accra	0.0827∗∗∗	0.0504∗∗∗	0.0882∗∗∗	0.0553∗∗∗
	[0.0483–0.142]	[0.0207–0.123]	[0.0512–0.152]	[0.0230–0.133]
Volta	0.466∗∗	0.185∗∗∗	0.367∗∗∗	0.174∗∗∗
	[0.279–0.779]	[0.0797–0.429]	[0.218–0.618]	[0.0762–0.400]
Eastern	0.392∗∗∗	0.377∗	0.373∗∗∗	0.402∗
	[0.234–0.654]	[0.175–0.811]	[0.224–0.621]	[0.194–0.832]
Ashanti	0.901	0.526	0.757	0.464∗
	[0.564–1.439]	[0.265–1.042]	[0.475–1.207]	[0.242–0.892]
Brong Ahafo	1	1	1	1
Northern	0.481∗∗∗	0.443∗	0.450∗∗∗	0.428∗∗
	[0.314–0.738]	[0.237–0.829]	[0.294–0.691]	[0.235–0.781]
Upper east	1.721∗	0.966	1.456	0.917
	[1.105–2.682]	[0.505–1.846]	[0.938–2.262]	[0.493–1.706]
Upper west	2.730∗∗∗	1.985∗	2.317∗∗∗	1.513
	[1.764–4.224]	[1.069–3.686]	[1.502–3.574]	[0.840–2.725]
*N*	10530	3300	8374	2812

Women and men, MICS 2011.

Odds ratios; 95% confidence intervals in brackets.

* *P < *0.05;

** *P < *0.01;

*** *P < *0.001.

Men aged 15–17 years were more likely to enrol in the NHIS than 18- to 24-year-old men (OR = 2.545, 99% CI: 1.609–4.024), similar to DHS results. The other age categories showed no statistically significant difference for men, whereas for women, the age groups 15–17 and 30–34 years were positively correlated with enrolment and statistically significant (OR = 1.349, 95% CI: 1.041–1.748 and OR = 1.309, 95% CI: 1.020–1.679, respectively).

People with higher wealth index levels were associated with higher enrolment rates for both men and women. Similarly, people with higher education levels were more likely to be registered in the NHIS.

MICS analyses showed that living in an urban setting was negatively correlated with the probability of registering with the NHIS (OR = 0.752, 95% CI: 0.586–0.965 for women and OR = 0.656, 95% CI: 0.435–0.988 for men). MICS regional results confirmed the DHS findings, with some exceptions. The relationship in the Volta region was negative and significant for both genders, we found a positive association in the Upper East region for women and Upper West for both genders. The relationship in the Ashanti region was not significant.

As with the DHS data, the MICS results obtained using the sample without exempted individuals did not highlight remarkable differences compared with the whole sample.

#### Ghana Living Standard Survey

In the GLSS analysis (Table 5), being married showed a positive and statistically significant relationship with enrolment only among men (OR = 1.433, 99% CI: 1.125–1.825). Women with children were more likely to be registered in the NHIS (OR = 1.504, 99% CI: 1.125–1.825), as well as pregnant women (OR = 3.09, 99% CI: 2.239–4.267).

**Table 5:** OR from a multilevel logistic regression

	Whole sample		Exempted excluded	
Prob. of holding a valid NHIS card	Women	Men	Women	Men
Married	1.241	1.433∗∗	1.198	1.454∗∗
	[0.944–1.632]	[1.125–1.825]	[0.886–1.619]	[1.101–1.921]
Male head	0.817	0.721∗	0.856	0.568∗∗
	[0.627–1.066]	[0.537–0.970]	[0.641–1.142]	[0.397–0.811]
Children	1.504∗∗∗	0.900	1.657∗∗∗	1.019
	[1.287–1.758]	[0.747–1.083]	[1.389–1.977]	[0.820–1.268]
Pregnant	3.091∗∗∗			
	[2.239–4.267]			
Age 15–17	1.246	1.656∗∗∗		
	[0.999–1.553]	[1.334–2.056]		
Age 18–24	1	1	1	1
Age 25–29	1.219	0.754∗	1.241	0.756∗
	[0.963–1.542]	[0.583–0.976]	[0.965–1.595]	[0.575–0.994]
Age 30–34	1.329∗	1.111	1.326∗	1.029
	[1.026–1.721]	[0.825–1.496]	[1.005–1.751]	[0.748–1.416]
Age 35–39	1.609∗∗∗	1.057	1.637∗∗∗	0.980
	[1.241–2.087]	[0.772–1.447]	[1.236–2.168]	[0.698–1.375]
Age 40–44	1.256	1.614∗∗	1.266	1.532∗
	[0.962–1.640]	[1.168–2.232]	[0.953–1.683]	[1.077–2.178]
Age 45–49	1.303	1.284	1.250	1.201
	[0.980–1.732]	[0.921–1.791]	[0.925–1.689]	[0.837–1.723]
Age 50–54	1.393∗	1.921∗∗∗	1.453∗	1.788∗∗
	[1.049–1.850]	[1.370–2.693]	[1.079–1.958]	[1.239–2.581]
Age 55–59	3.162∗∗∗	3.544∗∗∗	2.517∗∗∗	2.260∗∗∗
	[2.560–3.907]	[2.743–4.579]	[1.946–3.256]	[1.652–3.093]
lncome	1.498∗∗∗	2.123∗∗∗	1.688∗∗∗	2.475∗∗∗
	[1.291–1.738]	[1.826–2.468]	[1.432–1.991]	[2.074–2.954]
No education	1	1	1	1
Primary not completed	1.317	1.381∗	1.613∗∗	1.538∗
	[0.982–1.766]	[1.013–1.883]	[1.171–2.222]	[1.081–2.187]
Completed primary	1.283	1.128	1.555∗∗	1.355
	[0.953–1.728]	[0.821–1.548]	[1.125–2.150]	[0.948–1.938]
Lower secondary	2.040∗∗∗	2.151∗∗∗	2.670∗∗∗	2.822∗∗∗
	[1.588–2.621]	[1.657–2.793]	[2.027–3.517]	[2.085–3.821]
Upper secondary	3.329∗∗∗	2.839∗∗∗	4.095∗∗∗	3.876∗∗∗
	[2.281–4.859]	[1.978–4.075]	[2.721–6.162]	[2.587–5.808]
Post-secondary	4.293∗∗∗	5.103∗∗∗	5.524∗∗∗	6.515∗∗∗
	[2.779–6.633]	[3.331–7.818]	[3.454–8.834]	[4.016–10.57]
University and higher	5.116∗∗∗	6.170∗∗∗	5.860∗∗∗	8.368∗∗∗
	[3.200–8.179]	[3.969–9.591]	[3.539–9.702]	[5.097–13.74]
Literacy programme	1.663∗	1.437	1.731∗	1.209
	[1.088–2.540]	[0.918–2.249]	[1.084–2.765]	[0.717–2.039]
Other	6.013	0.542	7.630	0.337
	[0.162–223.0]	[0.0145–20.22]	[0.192–303.9]	[0.00329–34.43]
Not working	1.318∗	1.731∗∗∗	1.365∗	1.795∗∗∗
	[1.033–1.683]	[1.341–2.236]	[1.017–1.831]	[1.303–2.471]
A paid employee	1.454∗	1.877∗∗∗	1.489∗	2.177∗∗∗
	[1.052–2.008]	[1.458–2.415]	[1.046–2.118]	[1.633–2.904]
Non-agric s.e. with employees	1.531	1.043	1.477	1.194
	[0.980–2.390]	[0.659–1.651]	[0.911–2.395]	[0.728–1.960]
Non-agric s.e. without employees	1.389∗∗	1.148	1.327∗	1.275
	[1.091–1.768]	[0.850–1.549]	[1.013–1.740]	[0.912–1.782]
Non-agric contributing family worker	1.459	2.675∗∗∗	1.290	3.114∗∗
	[0.960–2.219]	[1.517–4.717]	[0.767–2.169]	[1.525–6.359]
Agric s.e. with employees	0.974	1.627	0.922	1.592
	[0.443–2.142]	[0.997–2.656]	[0.381–2.232]	[0.918–2.761]
Agric s.e. without employees	1	1	1	1
Agric contributing family worker	1.111	1.373∗	1.051	1.244
	[0.876–1.409]	[1.059–1.780]	[0.800–1.380]	[0.909–1.702]
Domestic employee	1.278	1.115	2.040	1.332
	[0.233–6.994]	[0.137–9.070]	[0.317–13.13]	[0.144–12.31]
Casual workers	0.599	0.697	0.447	0.860
	[0.291–1.232]	[0.396–1.227]	[0.198–1.006]	[0.460–1.606]
Apprentice	1.346	0.781	1.290	0.905
	[0.835–2.170]	[0.433–1.408]	[0.761–2.184]	[0.470–1.740]
Other	0.478	0.0992∗	0.571	0.201
	[0.0345–6.642]	[0.0102–0.965]	[0.0390–8.355]	[0.0175–2.292]
Morbidity	1.324∗∗	1.403∗∗	1.158	1.334∗
	[1.071–1.637]	[1.124–1.752]	[0.918–1.460]	[1.037–1.717]
Disability	0.843	1.212	0.861	1.268
	[0.570–1.247]	[0.823–1.787]	[0.520–1.424]	[0.764–2.104]
Urban	1.245	1.185	1.181	0.988
	[0.820–1.891]	[0.818–1.718]	[0.769–1.816]	[0.663–1.473]
Western	0.0343∗∗∗	0.0508∗∗∗	0.0382∗∗∗	0.0555∗∗∗
	[0.0146–0.0806]	[0.0238–0.109]	[0.0158–0.0921]	[0.0245–0.126]
Central	0.0930∗∗∗	0.106∗∗∗	0.0889∗∗∗	0.0957∗∗∗
	[0.0398–0.217]	[0.0497–0.227]	[0.0370–0.213]	[0.0420–0.218]
Greater Accra	0.0293∗∗∗	0.0316∗∗∗	0.0276∗∗∗	0.0290∗∗∗
	[0.0126–0.0684]	[0.0148–0.0674]	[0.0115–0.0664]	[0.0127–0.0660]
Volta	0.442	0.354∗∗	0.408∗	0.364∗
	[0.192–1.017]	[0.170–0.738]	[0.174–0.959]	[0.165–0.799]
Eastern	0.289∗∗	0.273∗∗∗	0.295∗∗	0.246∗∗∗
	[0.127–0.654]	[0.133–0.561]	[0.127–0.682]	[0.114–0.533]
Ashanti	0.0695∗∗∗	0.0820∗∗∗	0.0751∗∗∗	0.0856∗∗∗
	[0.0310–0.156]	[0.0399–0.168]	[0.0327–0.173]	[0.0394–0.186]
Brong Ahafo	1	1	1	1
Northern	0.243∗∗∗	0.361∗∗	0.297∗∗	0.490
	[0.106–0.559]	[0.174–0.748]	[0.127–0.697]	[0.225–1.067]
Upper east	6.447∗∗∗	4.807∗∗∗	8.061∗∗∗	6.683∗∗∗
	[2.717–15.30]	[2.250–10.27]	[3.304–19.67]	[2.949–15.15]
Upper west	2.834∗	4.099∗∗∗	3.696∗∗	6.103∗∗∗
	[1.186–6.776]	[1.914–8.778]	[1.505–9.079]	[2.686–13.87]
*N*	22 561	19 748	17 937	16 185

Women and men, GLSS 2012–13.

Odds ratios; 95% confidence intervals in brackets.

s.e., self-employed.

* *P < *0.05;

** *P < *0.01;

*** *P < *0.001.

Similar to DHS and MICS results, men under 18 years old were more likely to register than those in the referent (OR = 1.656, 99% CI: 1.334–2.056) and men between 25 and 29 years of age were less likely to register (OR = 0.754, 95% CI: 0.583–0.976). In contrast, we did not see differences among women in these age categories. Women aged 30–39 years and men and women aged 50–59 years were associated with higher enrolment rates than people aged 18–24 years.

Both men and women with higher education levels were more likely to be registered in the NHIS. Household expenditure, a proxy for income, also showed a statistically significant and positive correlation with NHIS enrolment. In another version of the model (data not shown), we found that the squared term of this income proxy was not significant, indicating insufficient evidence for a U-shaped relationship between household expenditure and enrolment.

The probability of enrolment in the NHIS varied by occupational status. Women and men registered as ‘paid employee’ (OR = 1.454, 95% CI: 1.052–2.008 and OR = 1.877, 99% CI: 1.458–2.415, respectively) or ‘not working’ (OR = 1.318, 95% CI: 1.033–1.683 and OR = 1.731, 99% CI: 1.341–2.236) were more likely to be enrolled in the NHIS than people who were agricultural self-employed without employees. For men, the two categories ‘contributing family worker’ (both agricultural and non-agricultural) were positively correlated with enrolment (OR = 1.373, 95% CI: 1.059–1.780 and OR = 2.675, 99% CI: 1.517–4.717, respectively) and for women as well, though results are not statistically significant. Finally, also the category ‘non-agricultural self-employed without employees’ showed a positive and significant association for women (OR = 1.389, 99% CI: 1.091–1.768). This was similar to the findings of the DHS analysis, which showed that people working in the agricultural sector had lower probability of enrolment, even though the categories considered in the GLSS and DHS analyses were slightly different.

The probability of NHIS enrolment for people living in urban or rural settings did not differ statistically. Regional results using the GLSS are consistent with what we found in the analysis with MICS data, with two exceptions. Volta region shows a negative but not significant results for women and Ashanti shows negative and significant results for both women and men, similar to DHS results.

In the GLSS analysis, we added two proxies of health status, namely having had a morbidity episode in the last 2 weeks and suffering from any form of physical or mental disability. Results suggested that the former was correlated with the probability of being enrolled in the NHIS (OR = 1.324, 99% CI: 1.071–1.637 for women and OR = 1.403, 99% CI: 1.124–1.752 for men), while the latter was not.

Finally, there are some slight differences in the results when we exclude the individuals who receive premium exemptions. Women with a low level of education [incomplete or completed primary school (OR = 1.613, 99% CI: 1.171–2.222 and OR = 1.555, 99% CI: 1.125–2.150, respectively)] are significantly more likely to enrol than women without education. Agricultural contributing family workers (men) report still a positive relationship, but not statistically significant. Morbidity is still significant for men but not for women.

## Discussion and conclusion

This study assessed the socio-economic factors associated with NHIS enrolment in Ghana. It is, to our knowledge, the first study that uses three national surveys to investigate this topic in Ghana.

More generally, this study offers a novel contribution to the literature on the socio-economic and demographic determinants of enrolment into social health insurance schemes in LMICs, accounting for contextual factors, and using nationally representative households surveys.

The structure of all the three databases allowed us to run multilevel logistic regression models where the first level is a small geographic area, the second level is the household and the third level is the individual. This econometric approach accounts for specific cluster effects indirectly related to contextual factors and for the correlation among household members. In this way, we did not only assess demand-side factors, but we also indirectly accounted for the heterogeneity of local health systems factors—e.g. availability and quality of health services—and other contextual factors—e.g. community enrolment rates, social supportive networks, etc.

The analyses of the three national surveys showed similar, albeit not identical, patterns of the determinants of NHIS enrolment. The study extends the existing evidence on the determinants of enrolment in the NHIS, providing some interesting new findings that may inform policy decisions and provide a baseline analysis for longitudinal analyses in Ghana, and raise research questions for similar investigations in other contexts. Similarly to what was found in previous studies (Chankova *et al.*, 2008; Sarpong *et al.*, 2010; Jehu-Appiah *et al.*, 2011; Dixon *et al.*, 2011, 2014; Boateng and Awunyor-Vitor, 2013; Kumi-Kyereme and Amo-Adjei, 2013; Owusu-Sekyere and Chiaraah, 2014; Duku *et al.*, 2015; Amo-Adjei *et al.*, 2016; Bonfrer *et al.*, 2016; Brugiavini and Pace, 2016; Kotoh and Van der Geest, 2016), higher wealth and education was associated with a higher probability of being enrolled in the NHIS. This was evident in particular in the analyses of GLSS and MICS data. Education is usually correlated with income and wealth, but it is also itself an explanation of higher enrolment, given that a more educated person is usually better informed.

Marital status was significantly associated with higher NHIS enrolment rates (with DHS data this held only for women) similarly to what found in a previous study (Boateng and Awunyor-Vitor, 2013), but differently from what was found in others recent manuscripts (Owusu-Sekyere and Chiaraah, 2014; Duku *et al.*, 2015; Amo-Adjei *et al.*, 2016). Our result may perhaps be due to the fact that married couples might be better able to afford insurance premiums than their single counterparts. For men, it might suggest that women play a role in the enrolment decision of their husbands. Secondly, in Ghana it is more likely that women enrol their children in the NHIS, rather than men. Many women may enrol themselves and their children at the same time, usually as soon as their children get sick, avoiding going to the registration office twice. Contrary to what was found by other studies (e.g. Jehu-Appiah *et al.*, 2011), there was insufficient evidence that rural or urban residence impacts NHIS enrolment. The analyses of DHS and GLSS data did not show differences in the probability of enrolling in the NHIS between rural and urban settings, while the analysis of MICS data indicated that the probability of being registered was weakly correlated with living in a rural area. We then found mixed results regarding the relationship between age and NHIS enrolment. Neither in the previous literature age was significantly correlated to NHIS enrolment for the majority of the studies but one (Blanchet *et al.*, 2012). The probability of being registered was higher for pregnant women and women with children, two groups of people—pregnant women and children—exempted from paying the premiums.

Another interesting and novel finding was that occupational field (only available in the DHS and GLSS surveys) had a significant impact on enrolment: women and men working in a professional sector, family workers and those in the service or retail sale sector and even unemployed were more likely to be enrolled in the NHIS than individuals employed in the agricultural sector. This result could be attributed to the fact that workers of agricultural sector cannot rely on a stable or predictable income, and possibly, that the burden of losing working time to go to the NHIS registration office presents a disincentive to enrol. In addition to these factors, the costs of seeking care (e.g. the transport costs to reach the NHIS enrolment office) might be high for people working in the agricultural sector who might be more likely to live in rural settings (Macha *et al.*, 2012). Another reason for low enrolment might be the lack of knowledge about the NHIS. Yet, there is limited evidence of these potential factors in the literature published so far; only one recent study by Bonfrer *et al.* (2016) revealed that women without occupation or working in agriculture were less likely to enrol in the NHIS and another study reveals a positive correlation between employment and enrolment (Gobah and Zhang, 2011).

As an additional sensitivity analysis (data not shown), we re-analysed the DHS and GLSS datasets and examined one additional available variable: i.e. the use of the outpatient services in the last two weeks (GLSS survey) or 6 months (DHS survey). As expected, results from both surveys showed that recent use of health care services was positively correlated with enrolment in the NHIS.

Since the information on the use of services and on enrolment is gathered simultaneously, it is generally impossible to assess causality using only these data. In particular, it is not possible to disentangle the potential adverse selection effect, defined as the phenomenon in which individuals enrol in a health insurance only if they expect that the benefit will outweigh the cost of premiums, from the moral hazard behaviour related to potential over use of services by NHIS members. However, when the use of health services was included in the GLSS regression model, suffering from a morbidity episode was no longer associated with higher enrolment rates, perhaps because attending at least one health care visit captures the effect of having had a morbidity episode. This finding, together with a positive and significant correlation of the morbidity variable when the use of service is not included, might support a negative self-selection problem rather than moral hazard behaviour.

This would be in line with the findings of other studies showing that the ex-ante moral hazard is unlikely to occur in the Ghanaian health system (Powell-Jackson *et al.*, 2014). Results obtained with GLSS data may provide some additional indication that health insurance enrolment is correlated with health care needs, the well-known adverse selection phenomenon. Similar evidence has already been found in the literature in the Ghanaian setting (Duku *et al.*, 2016), as well as in other LMIC settings (Zhang and Wang, 2008; Lammers and Warmerdam, 2010).

The study has some limitations, primarily arising from the nature of data available. First of all, the data’s cross-sectional nature precluded any causal analysis between the socio-economic factors and NHIS enrolment, but the analysis still serves as an important baseline with which to consider change over time in enrolment in future analyses of NHIS within the Ghanaian context. Second, because we only considered cross-sectional data at a single time-point, we could not examine how contextual factors at the national level interact with or modify the impact of household and individual factors on insurance enrolment, although our analysis was apt to examine regional, household, and individual factors and enrolment. The cross-sectional analysis, moreover, only captures whether a person was registered in the NHIS at the time of the interview but does not indicate how consistently the person maintains insurance coverage, allowing us to understand contact with, and awareness of, NHIS rather than the role of insurance coverage across the life-span—a question that would require a longitudinal (follow-up) study. Therefore, we caution against the assumption that individual and household characteristics of people who have never been enrolled resemble the characteristics of people with intermittent coverage. We emphasize that the dropouts subgroup warrants further investigation.

Thus far, most policies aimed at increasing the uptake of health insurance in LMICs have focused on identifying and targeting the poorest populations and/or certain age groups. Incentive programmes generally provide health insurance premium exemptions, monetary subsidies and conditional or unconditional cash transfers (Lagarde *et al.*, 2007; Cashin *et al.*, 2017). For example, the Livelihood Empowerment Against Poverty (LEAP) programme, one of the main social protection schemes in Ghana, provides cash and health insurance to extremely poor households in some areas of the country (Handa *et al.*, 2013). Unfortunately, specific information on enrolment in LEAP programme was not available in the databases analysed and the size of this programme is still limited for being analysed in nationally representative household surveys. Nevertheless, the results of this study suggest that subsidizing underprivileged populations might not be enough to increase NHIS enrolment; policies aimed at reducing the opportunity costs faced by informal workers may play an equally important role in boosting NHIS enrolment. In Ghana, it might require implementing innovative ways to facilitate enrolment, such as using temporary and portable registration offices close to the fields to assist farmers to register with the NHIS. 

The results of this study shed light on the complex dynamics and factors behind low NHIS enrolment rates, indicating that current policies might be insufficient to encourage broad health insurance enrolment and by extension, to meet the goals of UHC.

*Ethical approval.* The authors obtained the ethical clearance from the Ghana Health Service Ethics Review Committee (GHS-ERC 01/05/16) for a project titled ‘Health System Governance for an inclusive and sustainable social health protection in Ghana and Tanzania’ of which this study is part.

## Supplementary Material

czz079_Supplementary_Data
