# Supplementary material for: Determinants of health insurance enrolment in Ghana: evidence from three national household surveys
Source: Health Policy Plan. 2019 Aug 21;34(8):582–94. doi: 10.1093/heapol/czz079 (PMC6794569; doi:10.1093/heapol/czz079)
Supplement: czz079_Supplementary_Data [file czz079_supplementary_data.docx]

# Supplementary materials


## Variables included in the regression models

Table 6: Variable included in the regression models

Variable Description

Marital status This variable is equal to one if the person is married or living with a partner, zero if

she/he has never been in union, or is widowed, divorced or separated.

Age Categorical variable. Both MICS and DHS data interview women from 15 to 49 years old, and men between 15 and 59 years of age. The GLSS interviews individuals of 0 to 59 for both sexes. In order to be consistent and to avoid biasing the values of the other coefficients, we kept young people (0–14) out of the estimation in GLSS, but descriptive statistics include the whole sample.

Education Categorical variable. DHS and MICS have the same 4 categories (no education, primary education, middle education, secondary education), while in GLSS this vari- able is classified into 6 categories (no education, primary education not completed, completed primary education, lower secondary education, upper secondary education, post-secondary education, university and higher education).

Occupation Categorical variable. DHS breaks this variable down into eight categories: not work- ing, professionals, clerical, sales, agriculture, services, skilled manual, unskilled man- ual. MICS does not collect information on the type of occupation of people inter- viewed. GLSS data is divide occupation into the following categories: not working, paid employee, non-agricultural self-employed with employees, non-agricultural self- employed without employees, non-agricultural contributing family worker, agricultural self-employed with employees, agricultural self-employed without employees, agricul- tural contributing family worker, domestic employee, casual workers for DHS data.

Health status In the first two datasets there is no proper information on household member health status. The only information we could include in the analyses of the DHS dataset is on whether the person has ever been diagnosed hypertension.*a* MICS does not have any information on this. In GLSS there are two health status indicators: whether the person has had any illness episode in the last two weeks (morbidity) and if the person suffers from any disability.

Income DHS and MICS data do not have a measure of household income. In the analysis we use the wealth index (5 categories based on the household assets), which is generally well correlated with income. GLSS has been designed to collect information also on the economic status of households. It contains data on both households’ income and expenditures. We include the total household expenditures (in a logarithmic scale) which is generally a more reliable measure of income.

Pregnant Dummy variable equal to one if the woman is pregnant.

Children DHS: dummy variable equal to one if there is at least one child under 5 living in the household.

MICS: dummy variable equal to one if there is at least one child living in the household. GLSS: dummy variable equal to one if the person has at least one child (regardless from where the child lives).

*a*Another variable in DHS dataset that gives information on the individual health status is the presence of anemia. Due to the high number of missing values in the data, we decided not to include it in the final estimates. In any case this variable was not statistically significant and the main findings remained the same.

## Results with different dependent variable

In this section we show the main models with the same regressors but a different dependent variable. We have included also those who replied ”yes, card not seen” in the dependent variable. The most important results do not change with respect to the main estimates. Nevertheless, there are some small differences. In the analysis using DHS data (Table 9) marital status was significantly and positively correlated among men. There is evidence of an income gradient effect among women, evidence that middle education has a positive correlation among women, as well as evidence that hypertension has a positive and significant relationship with enrollment for both men and women. Regional results remain the same with the exceptions of the Volta region, which showed a negative and significant relationship with enrollment among women, and the Upper East region which shows no difference with other regions among women.

In the analysis with MICS data, the biggest differences were the presence of children at home (which was not significant in this sub-analysis), and male head-of-household (which was significant in this sub-analysis) (Table 10). These two variables are likely to be correlated.

Finally, the GLSS analysis shows that having a male head-of-household is significant and negative also for men (Table 11). Education shows a stronger gradient effect among men and results regarding the kind of occupation slightly change with respect to the main results.

Table 7: OR from a multilevel logistic regression. Women and men, DHS 2014.


Table 7: OR from a multilevel logistic regression. Women and men, DHS 2014.

| Probability of holding a valid NHIS card | WOMEN | MEN |
| --- | --- | --- |
| Married | 1.749∗∗∗  [1.472,2.079] | 1.406∗  [1.066,1.854] |
| Male head | 0.963 | 1.075 |
|  | [0.826,1.122] | [0.791,1.460] |
| Children <5 | 1.106∗∗ | 1.028 |
|  | [1.029,1.188] | [0.921,1.148] |
| Pregnant | 3.266∗∗∗  [2.530,4.217] |  |
| Age 15–17 | 1.520∗∗∗  [1.225,1.886] | 2.124∗∗∗  [1.531,2.947] |
| Age 18–24 | 1 | 1 |
|  | [1,1] | [1,1] |
| Age 25–29 | 1.321∗∗  [1.085,1.608] | 0.562∗∗∗  [0.400,0.790] |
| Age 30–34 | 1.332∗∗  [1.078,1.647] | 0.664∗  [0.452,0.974] |
| Age 35–39 | 1.434∗∗ | 1.256 |
|  | [1.151,1.785] | [0.849,1.857] |
| Age 40–44 | 1.316∗ | 1.138 |
|  | [1.042,1.662] | [0.762,1.699] |
| Age 45–49 | 1.465∗∗  [1.142,1.879] | 1.875∗∗  [1.224,2.873] |
| Age 50–54 |  | 1.924∗∗  [1.236,2.995] |
| Age 55–59 |  | 1.483 |
|  |  | [0.919,2.394] |
| Poorest | 1 | 1 |
|  | [1,1] | [1,1] |
| Poorer | 1.166 | 1.358 |
|  | [0.928,1.464] | [0.983,1.875] |
| Middle | 1.347∗ | 1.346 |
|  | [1.033,1.756] | [0.927,1.954] |
| Richer | 1.962∗∗∗  [1.443,2.669] | 2.343∗∗∗  [1.510,3.635] |
| Richest | 2.791∗∗∗  [1.958,3.978] | 3.638∗∗∗  [2.179,6.077] |
| No education | 1 | 1 |
|  | [1,1] | [1,1] |
| Primary education | 1.054 | 1.001 |
|  | [0.870,1.277] | [0.715,1.404] |
| Middle education | 1.535∗∗∗ | 1.561∗∗ |

|  | [1.276,1.848] | [1.148,2.124] |
| --- | --- | --- |
| Secondary education | 2.099∗∗∗  [1.640,2.686] | 2.563∗∗∗  [1.752,3.751] |
| Not working | 1.666∗∗∗  [1.356,2.048] | 2.377∗∗∗  [1.680,3.364] |
| Professional sector | 2.331∗∗∗  [1.640,3.314] | 2.671∗∗∗  [1.787,3.995] |
| Clerical sector | 2.011∗ | 1.865 |
|  | [1.069,3.783] | [0.855,4.068] |
| Sales | 1.514∗∗∗ | 1.126 |
|  | [1.248,1.838] | [0.755,1.680] |
| Agriculture | 1 | 1 |
|  | [1,1] | [1,1] |
| Services | 2.318∗∗∗ | 1.727 |
|  | [1.413,3.800] | [0.922,3.233] |
| Skilled manual | 1.529∗∗∗ | 0.940 |
|  | [1.211,1.930] | [0.687,1.286] |
| Unskilled manual | 1.382 | 1.084 |
|  | [0.826,2.314] | [0.769,1.529] |
| Hypertension | 1.550∗∗∗  [1.214,1.979] | 1.337∗∗  [1.077,1.659] |
| Urban | 0.939 | 0.962 |
|  | [0.741,1.189] | [0.701,1.320] |
| Western | 0.277∗∗∗  [0.181,0.424] | 0.276∗∗∗  [0.162,0.469] |
| Central | 0.108∗∗∗  [0.0693,0.167] | 0.195∗∗∗  [0.112,0.339] |
| Greater Accra | 0.0614∗∗∗  [0.0387,0.0976] | 0.0635∗∗∗  [0.0344,0.117] |
| Volta | 0.560∗∗ | 0.584 |
|  | [0.362,0.865] | [0.340,1.003] |
| Eastern | 0.304∗∗∗  [0.199,0.466] | 0.219∗∗∗  [0.127,0.376] |
| Ashanti | 0.137∗∗∗  [0.0887,0.210] | 0.487∗∗  [0.288,0.824] |
| Brong Ahafo | 1 | 1 |
|  | [1,1] | [1,1] |
| Northern | 0.242∗∗∗ | 1.258 |
|  | [0.155,0.379] | [0.729,2.171] |
| Upper east | 0.743 | 2.325∗∗ |
|  | [0.472,1.168] | [1.322,4.088] |
| Upper west | 0.651 | 1.304 |
|  | [0.409,1.034] | [0.739,2.302] |
| *N* | 9339 | 4348 |
| Odds ratios; 95% confidence intervals in brackets |  |  |

∗ *p <* 0*.*05, ∗∗ *p <* 0*.*01, ∗∗∗ *p <* 0*.*001

Table 8: OR from a multilevel logistic regression. Women and men, MICS 2011.


Table 8: OR from a multilevel logistic regression. Women and men, MICS 2011

| Probability of holding a valid NHIS card | WOMEN | MEN |
| --- | --- | --- |
| Married | 1.879∗∗∗ | 1.258 |
|  | [1.516,2.329] | [0.857,1.847] |
| Male head | 1.253∗ | 1.015 |
|  | [1.048,1.497] | [0.663,1.555] |
| Children at home | 1.213 | 1.304 |
|  | [0.989,1.489] | [0.998,1.704] |
| Pregnant | 2.624∗∗∗  [2.081,3.310] |  |
| Age 15–17 | 1.400∗∗  [1.103,1.776] | 2.057∗∗∗  [1.397,3.029] |
| Age 18–24 | 1 | 1 |
|  | [1,1] | [1,1] |
| Age 25–29 | 1.154 | 0.862 |
|  | [0.921,1.445] | [0.558,1.332] |
| Age 30–34 | 1.306∗ | 1.017 |
|  | [1.029,1.657] | [0.617,1.677] |
| Age 35–39 | 1.204 | 1.329 |
|  | [0.945,1.533] | [0.794,2.224] |
| Age 40–44 | 1.395∗ | 1.042 |
|  | [1.081,1.801] | [0.599,1.814] |
| Age 45–49 | 1.114 | 1.755∗ |
|  | [0.860,1.444] | [1.099,2.800] |
| Poorest | 1 | 1 |
|  | [1,1] | [1,1] |
| Poorer | 2.330∗∗∗  [1.832,2.963] | 2.335∗∗∗  [1.570,3.475] |
| Middle | 4.085∗∗∗  [3.022,5.523] | 2.453∗∗∗  [1.489,4.042] |
| Richer | 8.148∗∗∗  [5.787,11.47] | 4.304∗∗∗  [2.498,7.416] |
| Richest | 11.75∗∗∗  [7.871,17.55] | 10.76∗∗∗  [5.445,21.25] |
| No education | 1 | 1 |
|  | [1,1] | [1,1] |
| Primary education | 1.260∗ | 1.344 |
|  | [1.035,1.534] | [0.906,1.995] |
| Middle education | 1.877∗∗∗  [1.534,2.296] | 1.858∗∗∗  [1.294,2.667] |
| Secondary education | 4.506∗∗∗  [3.402,5.969] | 3.273∗∗∗  [2.130,5.030] |
| Urban | 0.784∗ | 0.882 |

|  | [0.616,0.998] | [0.610,1.276] |
| --- | --- | --- |
| Western | 0.272∗∗∗  [0.164,0.451] | 0.425∗  [0.209,0.862] |
| Central | 0.173∗∗∗  [0.112,0.269] | 0.228∗∗∗  [0.121,0.433] |
| Greater Accra | 0.106∗∗∗  [0.0632,0.176] | 0.123∗∗∗  [0.0580,0.260] |
| Volta | 0.451∗∗  [0.271,0.750] | 0.279∗∗∗  [0.131,0.595] |
| Eastern | 0.448∗∗ | 0.480 |
|  | [0.270,0.743] | [0.230,1.004] |
| Ashanti | 0.853 | 0.635 |
|  | [0.532,1.367] | [0.324,1.242] |
| Brong Ahafo | 1 | 1 |
|  | [1,1] | [1,1] |
| Northern | 0.577∗  [0.378,0.881] | 0.491∗  [0.269,0.900] |
| Upper east | 1.870∗∗ | 1.120 |
|  | [1.200,2.916] | [0.595,2.108] |
| Upper west | 3.820∗∗∗  [2.455,5.942] | 2.923∗∗∗  [1.573,5.430] |
| *N* | 10530 | 3300 |
| Odds ratios; 95% confidence intervals in brackets |  |  |
| ∗ *p <* 0*.*05, ∗∗ *p <* 0*.*01, ∗∗∗ *p <* 0*.*001 |  |  |


Table 9: OR from a multilevel logistic regression. Women and men, GLSS 2012-2013

| Probability of holding a valid NHIS card | WOMEN | MEN |
| --- | --- | --- |
| Married | 1.359∗∗  [1.077,1.714] | 1.835∗∗∗  [1.487,2.266] |
| Male head | 0.859 | 0.533∗∗∗ |
|  | [0.686,1.077] | [0.411,0.691] |
| Children | 1.217∗∗  [1.061,1.397] | 0.838∗  [0.709,0.989] |
| Pregnant | 4.893∗∗∗  [3.604,6.643] |  |
| Age 15–17 | 1.162 | 1.652∗∗∗ |
|  | [0.956,1.412] | [1.358,2.010] |
| Age 18–24 | 1 | 1 |
|  | [1,1] | [1,1] |
| Age 25–29 | 1.207 | 0.775∗ |
|  | [0.982,1.483] | [0.619,0.972] |
| Age 30–34 | 1.120 | 0.945 |
|  | [0.894,1.404] | [0.727,1.227] |
| Age 35–39 | 1.339∗ | 1.160 |
|  | [1.064,1.684] | [0.880,1.528] |
| Age 40–44 | 1.139 | 1.383∗ |
|  | [0.901,1.440] | [1.037,1.843] |
| Age 45–49 | 1.184 | 1.375∗ |
|  | [0.920,1.525] | [1.024,1.848] |
| Age 50–54 | 1.407∗∗  [1.095,1.809] | 1.664∗∗∗  [1.230,2.251] |
| Age 55–59 | 3.393∗∗∗  [2.806,4.103] | 4.081∗∗∗  [3.235,5.149] |
| lncome | 1.790∗∗∗  [1.577,2.031] | 2.277∗∗∗  [1.998,2.596] |
| No education | 1 | 1 |
|  | [1,1] | [1,1] |
| Primary not completed | 1.327∗  [1.031,1.710] | 1.574∗∗  [1.198,2.069] |
| Completed primary | 1.531∗∗∗  [1.191,1.969] | 1.325∗  [1.008,1.742] |
| Lower secondary | 2.500∗∗∗  [2.017,3.100] | 3.034∗∗∗  [2.408,3.821] |
| Upper secondary | 4.991∗∗∗  [3.581,6.956] | 4.863∗∗∗  [3.526,6.707] |
| Post secondary | 5.998∗∗∗  [4.058,8.866] | 10.21∗∗∗  [6.864,15.18] |
| University and higher | 10.32∗∗∗ | 13.84∗∗∗ |

|  | [6.728,15.84] | [9.162,20.91] |
| --- | --- | --- |
| Literacy program | 1.525∗  [1.045,2.224] | 1.505∗  [1.003,2.257] |
| Other | 6.309 | 1.868 |
|  | [0.242,164.7] | [0.0780,44.71] |
| Not working | 1.663∗∗∗  [1.340,2.063] | 2.595∗∗∗  [2.060,3.268] |
| A paid employee | 1.647∗∗∗  [1.240,2.189] | 1.962∗∗∗  [1.568,2.455] |
| Non agric self-employed with employees | 1.463 | 1.255 |
|  | [0.984,2.174] | [0.847,1.860] |
| Non agric self-employed without employees | 1.415∗∗ | 1.046 |
|  | [1.147,1.747] | [0.805,1.358] |
| Non agric contributing family worker | 1.277 | 2.846∗∗∗ |
|  | [0.892,1.829] | [1.730,4.683] |
| Agric self-employed with employees | 0.836 | 1.322 |
|  | [0.423,1.655] | [0.843,2.074] |
| Agric self-employed without employees | 1 | 1 |
|  | [1,1] | [1,1] |
| Agric contributing family worker | 1.002 | 1.412∗∗ |
|  | [0.811,1.237] | [1.116,1.785] |
| Domestic employee | 1.922 | 0.952 |
|  | [0.475,7.781] | [0.148,6.112] |
| Casual workers | 1.049 | 0.619∗ |
|  | [0.557,1.975] | [0.384,0.995] |
| Apprentice | 1.158 | 1.301 |
|  | [0.769,1.745] | [0.804,2.105] |
| Other | 3.504 | 0.0738∗∗ |
|  | [0.302,40.68] | [0.0105,0.518] |
| Morbidity | 1.325∗∗  [1.103,1.592] | 1.431∗∗∗  [1.177,1.740] |
| Disability | 0.796 | 1.060 |
|  | [0.559,1.134] | [0.743,1.513] |
| Urban | 2.069∗∗∗  [1.548,2.764] | 1.649∗∗∗  [1.247,2.180] |
| Western | 0.226∗∗∗  [0.128,0.397] | 0.159∗∗∗  [0.0921,0.275] |
| Central | 0.106∗∗∗  [0.0596,0.188] | 0.0927∗∗∗  [0.0526,0.163] |
| Greater Accra | 0.0518∗∗∗  [0.0292,0.0920] | 0.0493∗∗∗  [0.0282,0.0863] |
| Volta | 0.371∗∗∗  [0.211,0.653] | 0.329∗∗∗  [0.190,0.569] |
| Eastern | 0.427∗∗  [0.245,0.743] | 0.370∗∗∗  [0.217,0.631] |

| Ashanti | 0.297∗∗∗  [0.173,0.510] | 0.304∗∗∗  [0.180,0.513] |
| --- | --- | --- |
| Brong Ahafo | 1 | 1 |
|  | [1,1] | [1,1] |
| Northern | 0.190∗∗∗  [0.107,0.335] | 0.273∗∗∗  [0.158,0.472] |
| Upper east | 4.871∗∗∗  [2.673,8.875] | 4.050∗∗∗  [2.289,7.164] |
| Upper west | 6.735∗∗∗  [3.649,12.43] | 9.137∗∗∗  [5.099,16.37] |
| *N* | 22561 | 19748 |
| Odds ratios; 95% confidence intervals in brackets |  |  |
| ∗ *p <* 0*.*05, ∗∗ *p <* 0*.*01, ∗∗∗ *p <* 0*.*001 |  |  |
